# Expired CO_2_ Levels Indicate Degree of Lung Aeration at Birth

**DOI:** 10.1371/journal.pone.0070895

**Published:** 2013-08-12

**Authors:** Stuart B. Hooper, Andreas Fouras, Melissa L. Siew, Megan J. Wallace, Marcus J. Kitchen, Arjan B. te. Pas, Claus Klingenberg, Robert A. Lewis, Peter G. Davis, Colin J. Morley, Georg M. Schmölzer

**Affiliations:** 1 The Ritchie Centre, Monash Institute of Medical Research, Monash University, Melbourne, Australia; 2 Division of Biological Engineering, Monash University, Melbourne, Australia; 3 School of Physics, Monash University, Melbourne, Australia; 4 Department of Pediatrics, Leiden University Medical Centre, Leiden, Netherlands; 5 Department of Paediatrics, University Hospital of North Norway and Paediatric Research Group, Faculty of Health Sciences, University of Tromsø, Tromsø, Norway; 6 Department of Medical Imaging and Radiation Science, Monash University, Clayton, Australia; 7 Biomedical Engineering, University of Saskatchewan, Saskatoon, Canada; 8 Department of Radiology and Biomedical Imaging, Monash University, Melbourne, Australia; 9 Department of Newborn Research, The Royal Women’s Hospital, Melbourne, Australia; 10 Division of Neonatology, Department of Pediatrics, Medical University, Graz, Austria; Icahn School of Medicine at Mount Sinai, United States of America

## Abstract

As neonatal resuscitation critically depends upon lung aeration at birth, knowledge of the progression of this process is required to guide ongoing care. We investigated whether expired CO_2_ (ECO_2_) levels indicate the degree of lung aeration immediately after birth in two animal models and in preterm infants. Lambs were delivered by caesarean section and ventilated from birth. In lambs, ECO_2_ levels were significantly (p<0.0001) related to tidal volumes and CO_2_ clearance/breath increased exponentially when tidal volumes were greater than 6 mL/kg. Preterm (28 days of gestation; term = 32 days) rabbits were also delivered by caesarean section and lung aeration was measured using phase contrast X-ray imaging. In rabbit kittens, ECO_2_ levels were closely related (p<0.001) to lung volumes at end-inflation and were first detected when ∼7% of the distal lung regions were aerated. ECO_2_ levels in preterm infants at birth also correlated with tidal volumes. In each infant, ECO_2_ levels increased to >10 mmHg 28 (median) (21–36) seconds before the heart rate increased above 100 beats per minute. These data demonstrate that ECO_2_ levels can indicate the relative degree of lung aeration after birth and can be used to clinically assess ventilation in the immediate newborn period.

## Introduction

Infants, particularly premature infants, commonly suffer respiratory failure after birth and require breathing support because their airways are partially liquid-filled [1]. This restricts the onset of pulmonary gas exchange and delays the cardiovascular changes that underpin the transition to air breathing at birth [1]–[3]. An international consensus on neonatal resuscitation recommends the use of positive pressure ventilation if infants fail to initiate spontaneous breathing immediately after birth [4]. However, it is difficult to achieve a balance between providing adequate ventilation without causing lung damage [5].

Transcutaneous oxygen saturation (SpO_2_) and heart rate (HR) are important indicators of adequate ventilation in the delivery room [6], [7], which are further improved with respiratory function monitoring to measure gas flows and tidal volumes [8], [9]. However, these parameters provide little information on ventilation efficiency and the degree of gas exchange and provide limited feedback to guide clinical care when cardiorespiratory indicators fail to improve.

CO_2_ is produced in tissues as a by-product of oxidative metabolism, enters the blood and is eliminated from the body by diffusion across the alveolar epithelium before it is exhaled in the expired gas. As CO_2_ can only be present in expired gas if gas exchange has commenced, expired CO_2_ (ECO_2_) levels may indicate the degree and success of lung aeration and gas exchange. Indeed, colorimetric assessments of expired CO_2_ are commonly used to confirm correct endotracheal tube placement in newborn infants following intubation [10].

Low CO_2_ levels measured in arterial blood (PaCO_2_) upon arrival in the intensive care unit suggest that preterm infants are at risk of over-ventilation in the delivery room [11]. As a result, end-tidal CO_2_ levels have been used to estimate PaCO_2_ levels in newborn infants, with varying results [12],[13]. However, estimating PaCO_2_ levels from end-tidal CO_2_ values assumes that CO_2_ diffusion across the alveolar epithelium is not limited by low perfusion or gas exchange area [14]. Although a safe assumption in adults, low pulmonary perfusion and reduced gas exchange surface area caused by lung liquid retention, may limit gas exchange in newly born infants. Thus, rather than indicating PaCO_2_ levels, ECO_2_ levels may indicate the degree of aeration within the distal gas exchange regions of the lung immediately after birth. Our aim was to investigate whether ECO_2_ values indicate the degree of lung aeration immediately after birth and provide feedback on the success of pulmonary ventilation.

To investigate the relationship between ECO_2_ levels and the onset of ventilation, we measured ECO_2_ levels in lambs immediately after birth. To determine the relationship between lung aeration and ECO_2_ levels, we measured ECO_2_ levels in ventilated newborn rabbits during phase contrast (PC) X-ray imaging. PC X-ray imaging exploits the refractive index difference between air and water to generate high resolution images of the lung as it aerates after birth [15]–[20]. Lung gas volumes can be measured at any stage of a breath from the images [18], allowing corresponding measures of ECO_2_ levels and the volume of the preceding inflation. We also investigated whether measures of ECO_2_ are feasible in the delivery room and correlate with recordings of tidal volume during positive pressure ventilation in preterm infants.

## Materials and Methods

### Animal Experiments

The SPring-8 and/or Monash University’s Monash Medical Centre Committee A animal ethics committees approved all animal experiments. All imaging studies on rabbits were conducted in experimental hutch 3 of beam line 20B2, in the Biomedical Imaging Centre at the SPring-8 synchrotron in Japan [18], [21]–[23].

#### Lamb studies

Near term lambs at 139±1 (mean ± standard error of the mean; SEM) days of gestation (term = 147 days) were exposed by hysterotomy, had carotid artery and jugular vein catheters inserted and were intubated with a cuffed endotracheal tube (size 4.5 mm). Lambs were delivered by caesarean section, dried, weighed, and placed under a radiant heater to maintain body temperature, infused with dextrose 50 mg/ml (i.v.) and lightly sedated (alfaxane iv 5–15 mg/kg/h; Jurox, Auckland, New Zealand). All lambs (n = 9) were ventilated (Dräger Babylog 8000plus, Lübeck, Germany) with a peak inflating pressure (PIP) of 35 cmH_2_O, positive end expiratory pressure (PEEP) of 5 cmH_2_O and fraction of inspired oxygen (FiO_2_) of 0.21. Five lambs were ventilated at 60 inflations/min (inspiratory times 0.5 s) and 4 initially received five, 3 s inflations separated by a 1 s expiratory phase, before switching to 60 inflations/min. After 10 minutes of ventilation, all lambs received volume-guarantee ventilation for 20 min, with a set tidal volume (V_T_) of 8 mL/kg. Lambs were euthanized by an overdose of sodium pentobarbitone at the end of the experiment. Heart rate (HR), mean arterial pressure (BP), V_T_, PIP and ECO_2_ levels were measured continuously and recorded at 1000 Hz using Powerlab (ADInstruments, Sydney, Australia). ECO_2_ levels were measured using a Novametrix mainstream CO_2_ analyzer (Capnogard, Novametrix, Wallingford, CT, USA). Every inflation was analyzed over the first 30 s of ventilation, after which 3 inflations were analyzed every 30 s for the remainder of the experiment; ∼200 inflations were analyzed per animal. CO_2_ clearance per inflation (mL/kg) was calculated by integrating the product of the instantaneous gas flow and ECO_2_ concentration throughout expiration. Arterial blood samples were collected to measure partial pressure of oxygen (PaO_2_), PaCO_2_ and pH (ABL30, Radiometer, Copenhagen, Denmark).

#### Rabbit studies

Pregnant (28 days of gestation; term = 32 days) New Zealand white rabbits (n = 5) were anaesthetized (propofol; i.v.; 12 mg/kg bolus), intubated and anaesthesia was maintained by isoflourane inhalation (1.5–4%). Kittens (n = 17) were exposed by caesarean section, sedated (Nembutal; 0.1 mg, i.p.) and intubated before they were delivered and positioned upright in a prewarmed (37°C) water-filled plethysmograph (head out) located in the path of the X-ray beam [15]–[20]. Kittens were ventilated with a custom-built ventilator [23], synchronized with image acquisition, using air and a V_T_ of 8 mL/kg (determined manually from the plethysmograph), PEEP of 8 cmH_2_O at 24 inflations/min for 7 minutes. Lung gas volumes (measured by plethysmography), airway pressures and a trigger signal indicating image acquisition were recorded digitally (Powerlab, ADInstruments; Sydney, Australia). Following the experiment, PIP and PEEP were varied to observe the resulting changes in ECO_2_ levels before kittens were killed with sodium pentobarbitone (>100 mg/kg; i.p.).

ECO_2_ levels were measured using a small mainstream CO_2_ analyzer placed in the expiratory limb of the ventilation circuit, immediately distal to the “Y” piece connecting to the endotracheal tube. Although small (∼1×1×0.5 cm^3^), the CO_2_ analyzer could not be incorporated into the “Y” piece due to its size and dead space volume (∼300 µL). As V_T_ of these kittens is only 150–200 µL, we determined the response delay between exhalation and CO_2_ detection. The volume of an infused gas (containing CO_2_) required to detect 10%, 50% and 100% of the CO_2_ level in our system was 380, 780 and 1280 µL, respectively. Thus, to synchronize the changes in CO_2_ levels with other respiratory changes, the ECO_2_ recording was retrospectively time shifted by the time required to expire 780 µL.

PC X-ray imaging was performed as previously described [15]–[20]. The X-ray energy was 24 keV and kittens were located 3.0 m upstream of a fibre optics CCD camera (Hamamatsu, C9300-124F), which provided an effective pixel size of 16.2 µm and an active field of view of 32(H) × 32(W) mm^2^. Image acquisition was synchronized with ventilation such that 7 images, 300 ms apart, were acquired during each respiratory cycle with an exposure of 40 ms. An output trigger signal indicated on the physiological recording the precise timing of each image acquisition. The PC X-ray images were used to measure lung gas volumes, using a phase retrieval analysis [18], [22]. Lung gas volumes were calculated from each image to calculate functional residual capacity (FRC), dead space volumes of the lung [17], end-inflation gas volumes at which CO_2_ could be first detected in the expired air and to define the relationship between end-inflation gas volumes and ECO_2_. To determine the relationship between ECO_2_ levels and end-inflation lung volumes in the absence of other variables, such as the number of inflations required to achieve adequate lung aeration, the measured lung volumes were group and compared with the ECO_2_ level measured for each inflation.

### Studies of Preterm Infants

All infants were born at The Royal Women’s Hospital, Melbourne, Australia, a tertiary perinatal centre where ∼ 6000 infants are delivered and more than 100 infants with a birth weight of <1000 g are admitted to the neonatal intensive care unit annually. The infants were enrolled in a randomized control trial comparing mask ventilation guided by either respiratory function monitoring or clinical assessment alone [9]. The trial was approved by The Royal Women’s Hospital Research and Ethics Committees and registered with Australian and New Zealand Clinical Trials Registry ACTRN12608000357358 [9]. Written consent was obtained before birth if the mother was not in established labour and if time permitted. Where this was not possible, a waiver of prospective consent was granted by the Research and Ethics Committees in accordance with Australian National Health and Medical Research Council guidelines and written parental consent was later obtained to use the data collected in the delivery room. Consent was sought from the parents of these infants, to use data obtained, as soon as possible after the birth.

Mask ventilation was provided with a size 00 round silicone face mask (Laerdal, Stavanger, Norway) connected to a T-piece device (Neopuff Infant Resuscitator, Fisher & Paykel Healthcare, Auckland, New Zealand), a continuous flow, pressure-limited device with a manometer and a PEEP valve. The default settings were a gas flow of 8 L/min, PIP of 30 cmH_2_O and PEEP of 5 cmH_2_O. A respiratory function monitor (Florian Acutronic Medical Systems AG, Hirzel, Switzerland), measured gas flow, airway pressure and inspiratory and expiratory V_T_ by integrating the flow signal [8]. Continuous ECO_2_ levels were measured using a mainstream CO_2_ monitor (Capnogard, Novametrix, Wallingford, CT, USA) placed between the T-piece and facemask. Gas flow, V_T_, airway pressure and ECO_2_ levels were recorded at 200 Hz using a dedicated computer with Spectra software (Grove Medical, London, UK). In preterm infants the pressure, flow, V_T_ and CO_2_ waveforms for each inflation were analyzed. If during facemask ventilation, the infant’s heart rate and oxygen saturations remained low, as determined by the attending clinician, the infant was intubated using a non-cuffed endotracheal tube. No matter whether the infant was intubated or ventilated via a facemask, the expired CO_2_ and V_T_ values were included for analysis when the assessed leakage was <30%_._

### Statistical Analysis

Results from animal experiments are presented as mean ± SEM. Results from studies in infants are presented as mean (standard deviation) or median (interquartile range; IQR). To relate V_T_ and end-inflation lung volumes with ECO_2_ values in lambs and rabbit kittens, maximum ECO_2_ values were clustered into groups based on the expired volume of the preceding inflation and a sigmoidal, 3 parameter, non-linear regression analysis performed. Similarly, to determine the relationship between CO_2_ clearance per breath and Vt, an exponential growth, 4 parameter non-linear regression analysis was performed. Differences with p<0.05 indicate a significant difference.

## Results

### Animal Experiments

#### Lamb studies

Compared to conventional ventilation (at 60 inflations/minute), initiating ventilation with five, 3 sec, inflations had no effect on any cardiorespiratory parameters examined. As a result, data from all lambs were combined to relate ECO_2_ to expired V_T_. Blood gas parameters measured at the beginning (fetal) and end of these experiments are presented in Table 1.

**Table 1:** Arterial blood gas parameters in lambs.

Blood gasparameter (n = 9)	Fetal	30 mins afterventilation onset
SaO_2_ (%)	56.0±6.1	79.9±6.5
pH	7.22±0.02	7.22±0.04
PaCO_2_ (mmHg)	53.7±5.8	47.3±2.5
PaO_2_ (mmHg)	28.3±4.9	44.8±2
Haemoglobin (g/dL)	11.5±0.48	12.6±0.5

**Figure 1:**
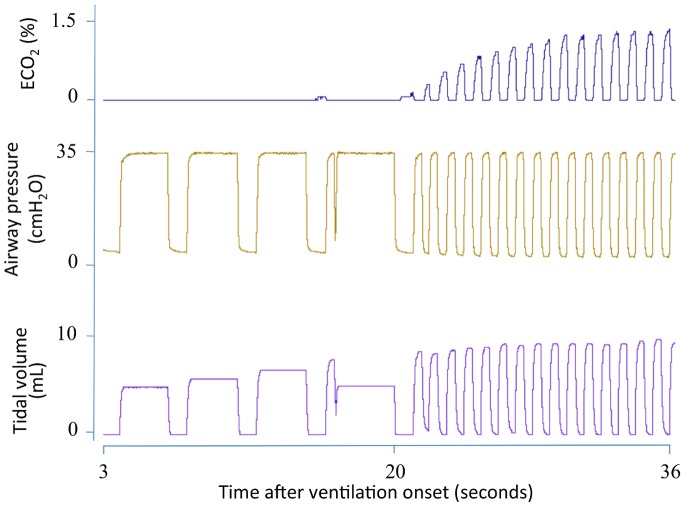
Expired CO_2_ levels, airway pressure and tidal volumes in a newborn lamb immediately after birth. Expired CO_2_ levels (ECO_2_) are demonstrated in the top panel, airway pressure in the middle panel and tidal volumes (V_T_) in the bottom panel. The lamb was resuscitated with five 3 second inflations (last 4 shown) followed by tidal ventilation. The first 3 inflations yielded no ECO_2_, despite achieving a V_T_ of 5–7 mL, whereas the 4^th^ and 5^th^ inflations produced small increases in ECO_2_ levels. Subsequent inflations produced a gradual increase in both ECO_2_ levels and V_T_.

**Figure 2:**
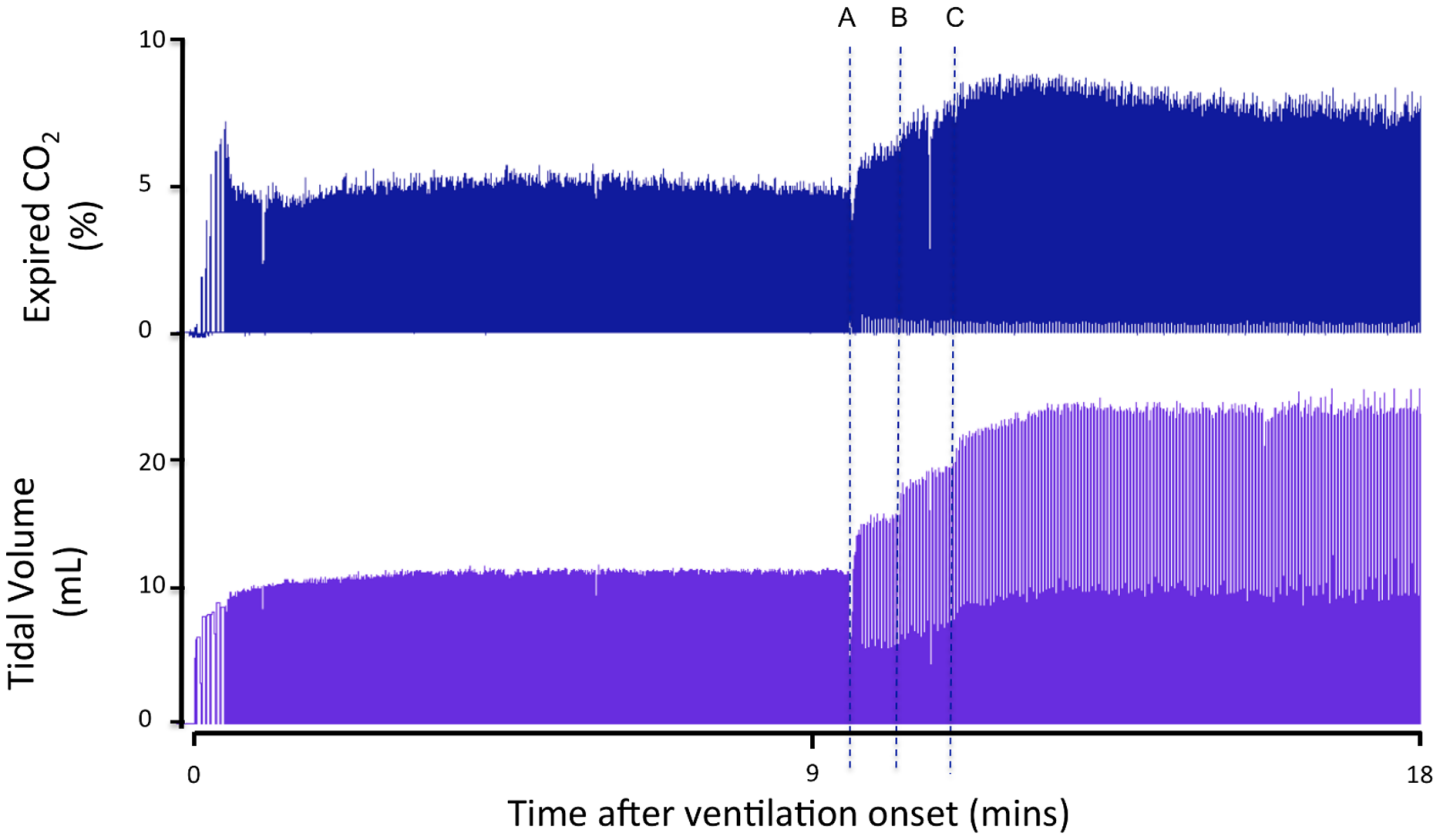
Expired CO_2_ levels and tidal volumes measured in a newborn lamb during the first 18 minutes after birth. Expired CO_2_ levels (ECO_2_) are demonstrated in the top panel and tidal volumes (V_T_) in the bottom panel. Tidal volumes were increased at “A” and “B” by increasing the inflation pressure and then the ventilation mode was changed to volume guarantee at “C” to target a V_T_ of 8 mL/kg. ECO_2_ levels and V_T_ were closely associated.

Figure 1 is a recording of the first 20 inflations in a lamb receiving 5 initial inflations of 3 s (first inflation not displayed) followed by ventilation at 60 inflations per minute. CO_2_ was not detected in the expired air of the first 3 inflations, despite achieving V_T_’s between 1.5 to 2 mL/kg, whereas both the 4^th^ and 5^th^ inflations (achieving >2 mL/kg) resulted in small increases in ECO_2_. With the next 15 inflations, at the same PIP, the ECO_2_ concentration rapidly increased (Fig. 1). Figure 2 shows the first 18 minutes of ventilation in a different lamb demonstrating the relationship between V_T_ and maximum ECO_2_ levels.

**Figure 3:**
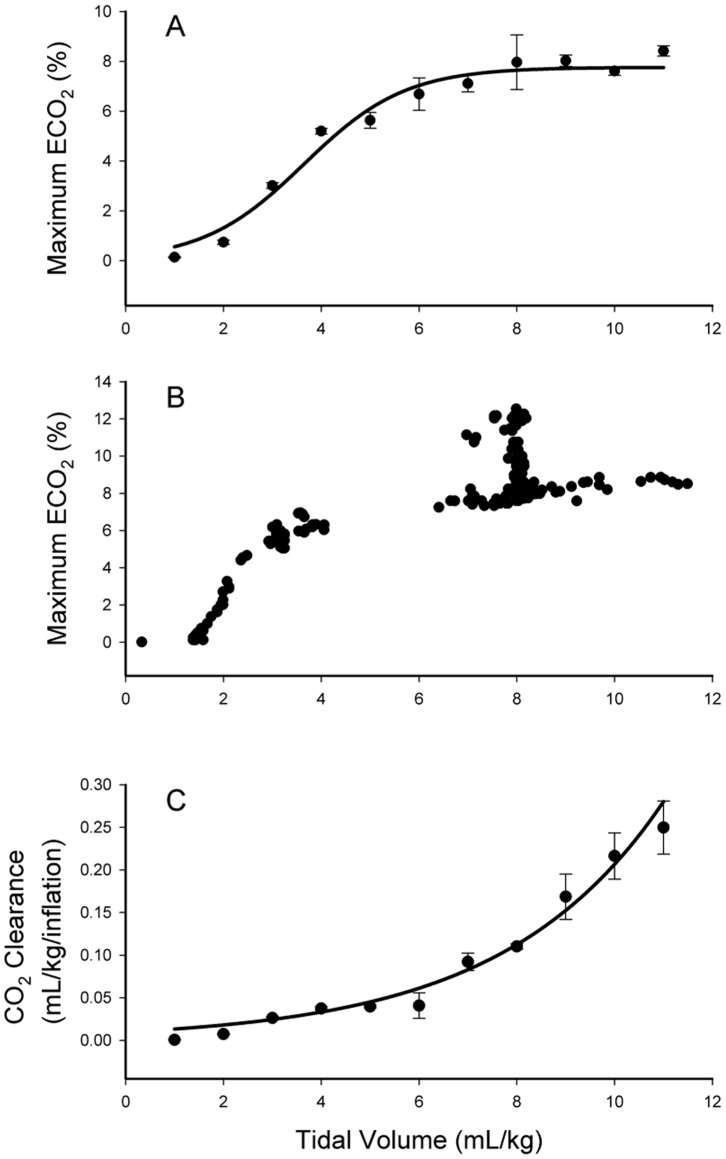
Relationship between expired CO_2_ levels at end-expiration and tidal volumes in lambs.

When data from all lambs were combined, maximum ECO_2_ levels were related to V_T_ after ventilation onset, following a sigmoidal relationship (p<0.0001) (Fig. 3A). The upper inflection point of the curve occurred at ∼6 mL/kg, indicating that V_T_ of at least 6 mL/kg were required to efficiently ventilate the lung. Data from an individual lamb are presented in Figure 3B, demonstrating the characteristic relationship between ECO_2_ levels and V_T_. At a V_T_ of 8 mL/kg, the set V_T_ after 10 min of ventilation, the ECO_2_ values tended to align vertically showing a large range at this volume. This reflects a gradual reduction in PaCO_2_ levels during the 20 mins of volume-controlled ventilation (decreased from 72.8 to 47.5 mmHg), resulting in a reduction in ECO_2_ levels for a set volume. CO_2_ clearance per inflation (Fig. 3C) was exponentially related (p<0.0001) to the V_T_, demonstrating a marked increase in CO_2_ clearance per breath at V_T_ >6 mL/kg in these lambs. In comparison, at V_T_ <6 mL/kg, the relationship between CO_2_ clearance and V_T_ was reduced, such that a doubling in V_T_ from 3 to 6 mL/kg had little effect on CO_2_ clearance; CO_2_ clearance per breath increased from 0.03±0.01 to 0.04±0.01 mL/kg/inflation.

#### Rabbit studies

Plethysmograph recordings and PC X-ray images were acquired from 17 preterm (28 days of gestation) newborn rabbits mean weight of 28.9±1.5 g.

**Figure 4:**
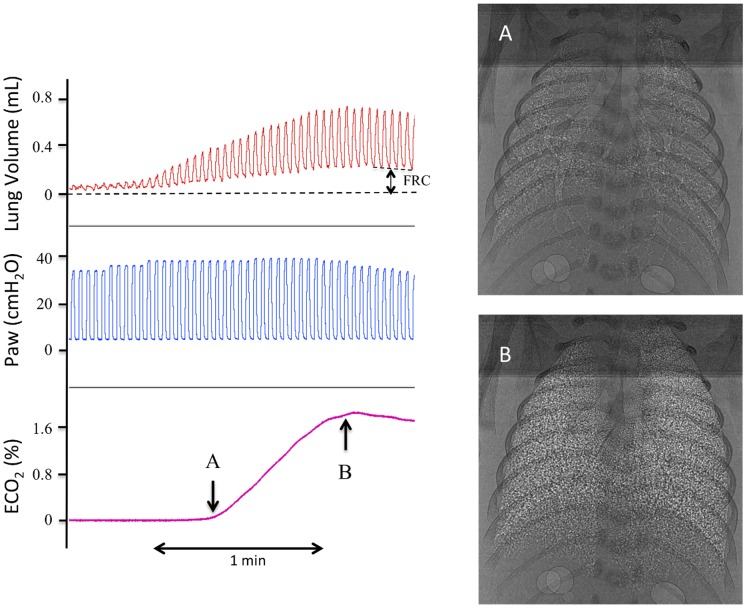
Lung gas volumes, airway pressures and expired CO_2_ levels in a ventilated newborn rabbit delivered preterm at 28 days of gestation. Lung gas volumes are demonstrated in the top panel, airway pressures in the middle panel and expired CO_2_ (ECO_2_) levels in the bottom panel. Images A and B were acquired at the times indicated by the arrows. The increase in ECO_2_ levels closely followed the increase in end-inflation lung volumes. All of the corresponding phase contrast X-ray images have been compiled into Movie S1.

**Figure 5:**
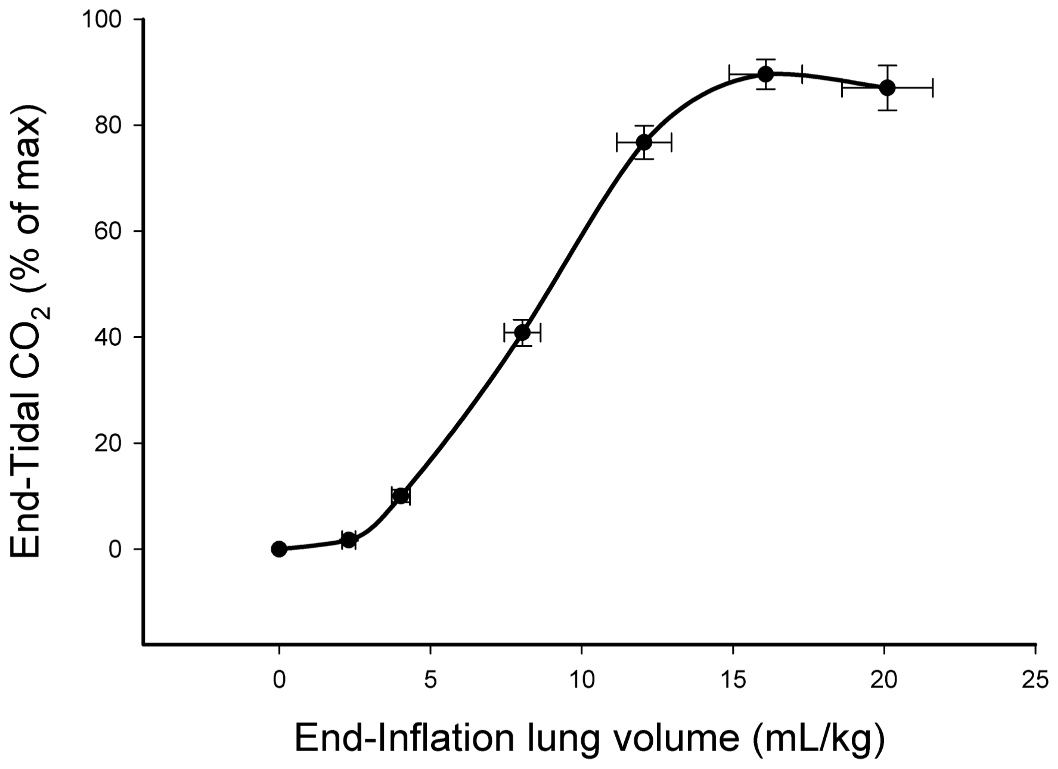
Relationship between expired CO_2_ levels and end-inflation lung gas volumes in ventilated newborn rabbits delivered preterm at 28 days of gestation. All data points are mean ± SEM and the relationship was highly significant (p<0.001), with an r^2^ value of 0.93.

Increasing ECO_2_ levels were closely associated with increasing V_T_ (Fig. 4) and relative aeration of the lung (see Movie S1), with the first appearance of gas in the distal airways almost exactly coinciding with the first detection of ECO_2_ (Fig. 4). The calculated dead space volume was 2.0±0.5 mL/kg and the end-inflation lung volume at which CO_2_ was first detected in expired gas was 3.4±0.3 mL/kg. The mean end-inflation lung gas volume (sum of FRC and V_T_) achieved was 20.1±1.5 mL/kg. The relationship between ECO_2_ levels and the immediate preceding end-inflation lung gas volume was highly significant (p<0.001; Fig. 5). Expressed as a percentage of the maximum ECO_2_ level achieved by each kitten, ECO_2_ levels increased from 0% at an end-inflation lung volume of 2.3±0.2 mL/kg to 40.8±2.5% at 8.0±0.6 mL/kg and to 89.6±2.8% at an end-inflation lung volume of 16.1±1.2 mL/kg (Fig. 5).

**Figure 6:**
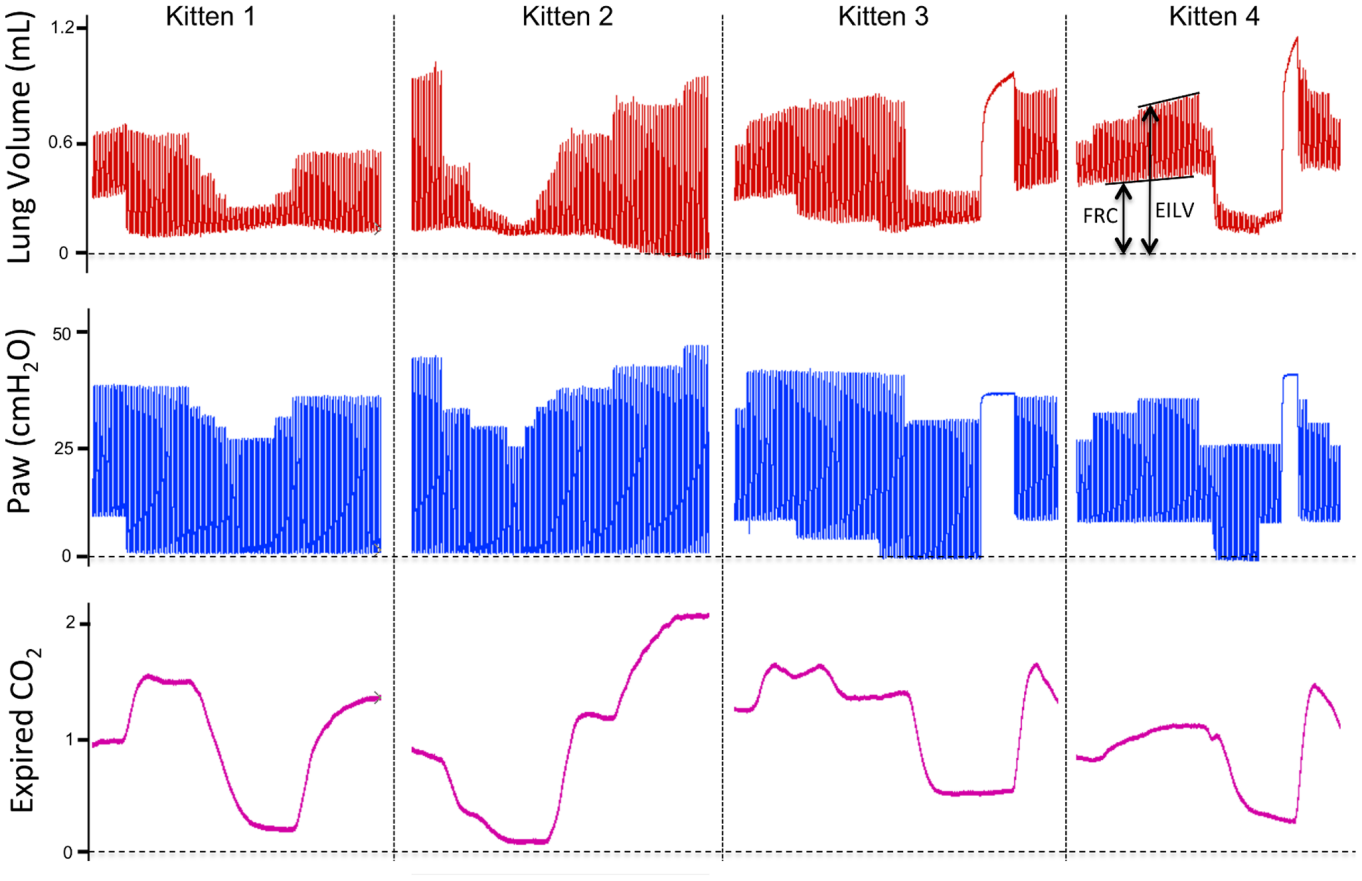
Lung gas volumes, airway pressures and expired CO_2_ levels in 4 ventilated newborn rabbits delivered preterm at 28 days of gestation. Lung gas volumes are demonstrated in the top panel, airway pressures in the middle panel and expired CO_2_ (ECO_2_) levels in the bottom panel. Functional residual capacity (FRC) and end-inflation lung volumes (EILV) are indicated on the lung volume trace. Changes in ECO_2_ levels closely followed changes in end-inflation lung volumes caused by altering ventilation parameters. The corresponding phase contrast X-ray images, compiled into a movie, for kitten 4 are displayed in Movie S2.

To identify the relative contribution of V_T_ and FRC (basal volumes at end-expiration; Fig. 6) to the relationship between end-inflation volumes and ECO_2_ levels, ventilation parameters were altered to determine the effect on ECO_2_ levels; 4 examples, from 4 different kittens, are displayed in figure 6. Increasing or decreasing end-inflation volumes always resulted in an increase or decrease in ECO_2_ levels, respectively (Fig. 6). The relationship between FRC and ECO_2_ levels was not significant (data not shown) as reductions in ECO_2_ were associated with both increases and decreases in FRC, depending on the associated V_T_ and end-inflation lung volumes. Reductions in FRC resulted in increased ECO_2_ levels, when associated with increased V_T_ (kittens 1 and 2), or reductions in ECO_2_ levels (kittens 3 and 4) when V_T_’s were reduced. V_T_ was significantly (p<0.05) associated with ECO_2_ levels (data not shown), although increasing V_T_ was not always associated with increasing ECO_2_ levels, particularly if end-inflation volumes were not altered. This occurred in kitten 3 (Fig. 6, first half of trace), where a reduction in FRC resulted in increased V_T_ and reduced ECO_2_ levels without a change in end-inflation volumes. The changes in lung gas volumes caused by altering ventilation parameters in kitten 4 are displayed in Movie S2.

### Preterm Infant Studies

**Figure 7:**
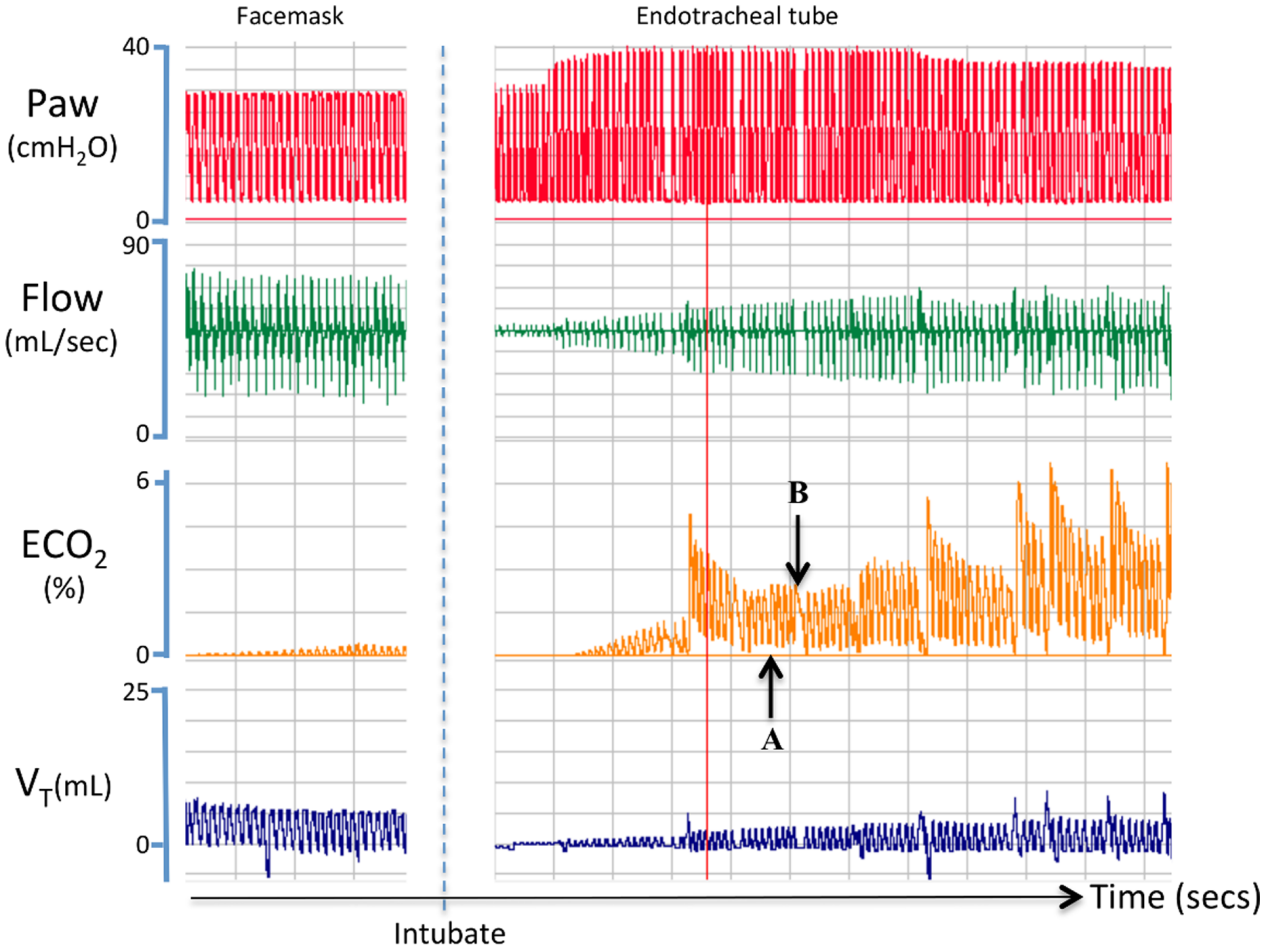
Airway pressure, gas flow, expired CO_2_ levels and tidal volumes measured in a preterm infant during positive pressure ventilation. This preterm infant received positive pressure ventilation that was initially applied via facemask and then followed by intubation. Before intubation, little expired CO_2_ (ECO_2_) could be detected, despite a good tidal volume (V_T_) and no detectable facemask leak. Following intubation, ECO_2_ levels rapidly increased with increasing V_T_ and preceded the increase in heart rate, which increased from 75 beats/min at “A” to 100 beats/min at “B”.

Demographics of the ten preterm infants included in the study are presented in table 2. Mask ventilation started at a median (IQR) time of 46 (37–60) seconds after birth. Time to achieve an ECO_2_ of >10 mmHg (1.3%) was 92 (46–150) seconds. Median (IQR) time for heart rate to exceed 100 beats/min was 126 (96–160) seconds and followed the increase in ECO_2_ levels to >10 mmHg by 28 (21–36) seconds (Table 3). Data recorded from a preterm infant during mask ventilation, followed by intubation, is presented in figure 7. During mask ventilation, despite an apparent good V_T_ with no leak, the infant remained bradycardic (68–72 beats/min) and very little ECO_2_ could be detected. Following intubation, the same PIP resulted in a markedly lower V_T_, necessitating an increase in PIP (to 40 cmH_2_O). The resulting increase in V_T_ was associated with increasing ECO_2_ levels, despite initially measuring smaller V_T_’s than during mask ventilation (Fig. 7). The measured HR and SpO_2_ values at intubation were 67 beats/min and 22%, respectively. Following restoration of PPV, an increase in PIP and a resulting increase in V_T_, ECO_2_ levels markedly increase, which was followed by an increase in the infant’s heart rate; increased from 70 beats/min at “A” to 100 beats/min at “B”.

**Table 2:** Demographics of included preterm infants.

Preterm infants (n = 10)	
Birth weight (gram)*	902 (287)
Gestational age (weeks)*	27 (2)
Male	5 (50%)
Apgar Score at 1 Minutes^#^	3 (2–4)
Apgar Score at 5 Minutes^#^	6 (5–6)
Infants Intubated	6 (60%)
Antenatal Steroids	10 (100%)
Caesarean Section	9 (90%)

Values are numbers (percentage) unless indicated,

* mean (standard deviation), ^#^median (interquartile range).

**Table 3:** Parameters of ECO_2_, HR at start of mask ventilation in preterm infants.

Preterm infants (n = 10)	
Time from birth to start mask ventilation (seconds)	51 (27–91)
Time to ECO_2_>10 mmHg (seconds)	96 (46–150)
Time to HR>100 beats/min (seconds)	126 (96–160)
Time between ECO_2_>10 mmHg and HR>100/min (seconds)	28 (21–36)
Speed of HR change (bpm/second)	45 (14–97)

Values are median (interquartile range).

**Figure 8:**
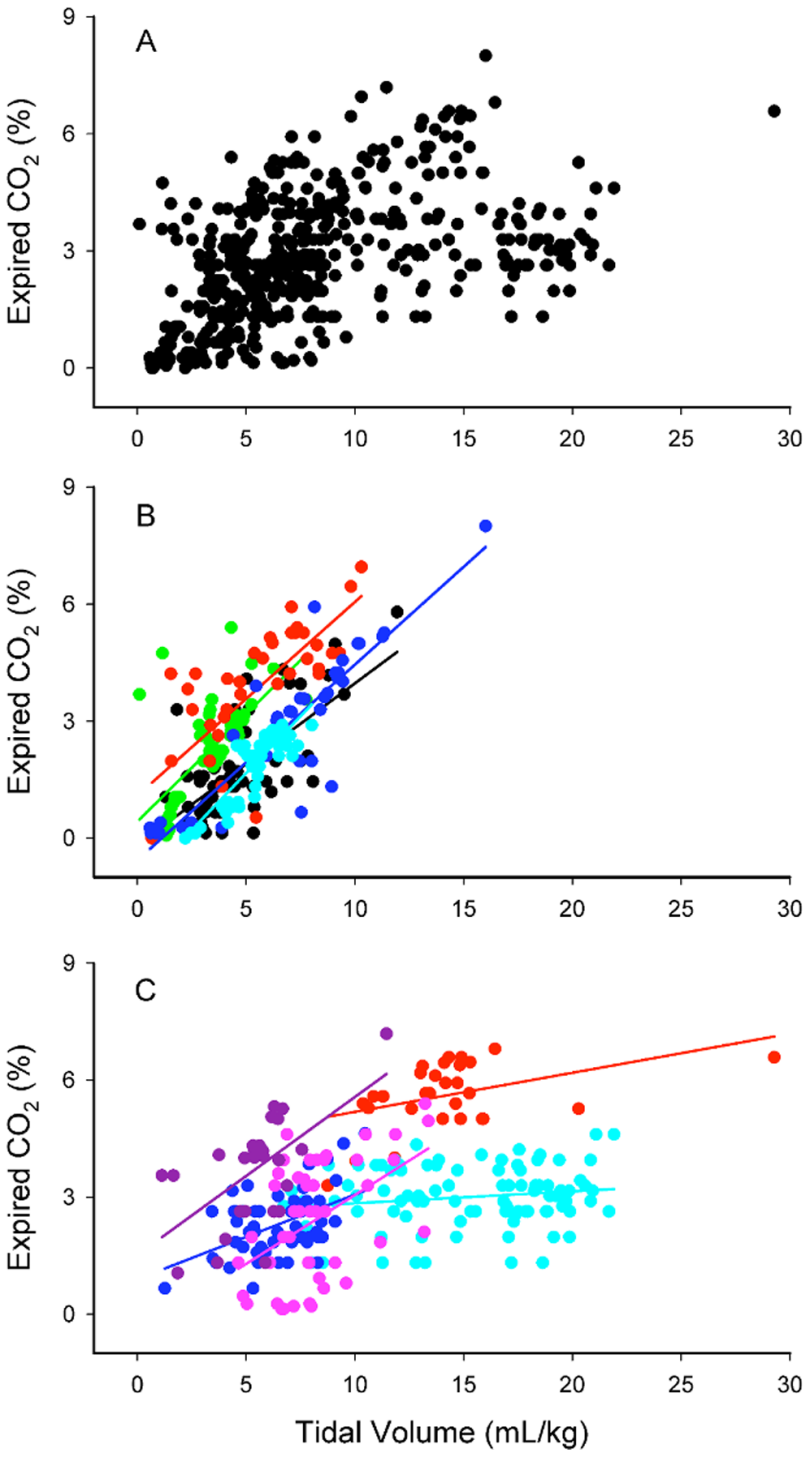
Maximum expired CO_2_ levels against tidal volume in preterm infants. A demonstrates data from all preterm infants studied (A; n = 10 infants and 517 breaths). The individual relationships between ECO_2_ and V_T_ for each infant are displayed in (B) and (C) for clarity.

Valid ECO_2_ and V_T_ data from all infants and inflations analyzed are presented in figure 8A. A significant (p<0.001) relationship between V_T_ and ECO_2_ levels (n = 10 babies, 517 breaths) was observed with the maximum ECO_2_ value increasing, on average, 0.15% with each 1 mL/kg increase in V_T_. As the relationship between ECO_2_ and V_T_ appeared to differ between infants, the relationship for each individual infant has been plotted in figures 8B (n = 5 infants) and 8C (n = 5 infants). All 5 infants in figure 8B showed a strong relationship between ECO_2_ values and V_T_.

## Discussion

We have demonstrated that ECO_2_ levels can provide important information to guide resuscitation immediately after birth when respiratory support fails to improve cardiorespiratory parameters in preterm infants. In the absence of mask leak, an inability to detect ECO_2_ indicates that gas has not penetrated down into the distal gas-exchange structures of the lung to allow gas exchange to commence. Increasing ECO_2_ levels in subsequent inflations indicate increasing aeration of distal gas exchange regions. An increase in ECO_2_ levels with increasing lung aeration was most closely associated with end-inflation lung volumes, was associated to a lesser degree with V_T_ and was not directly associated with FRC. These data confirm that ECO_2_ levels can indicate relative lung aeration immediately after birth but do not provide an accurate measure of PaCO_2_ levels. This is most likely due to limitations in gas exchange surface area and pulmonary perfusion [14].

As CO_2_ is highly soluble and the gas exchange surface area is large in a mature lung, the primary factors regulating end-tidal CO_2_ levels are the CO_2_ partial pressure gradient between alveolar gas and blood and the rate of pulmonary perfusion. As such, end-tidal and mixed venous CO_2_ levels can be used to estimate pulmonary perfusion and cardiac output [14]. In the neonate immediately after birth, although both pulmonary perfusion and CO_2_ partial pressure gradients likely influence ECO_2_ levels once gas exchange commences, our data indicate that the degree of lung aeration at end inflation (when gas volumes and gas exchange surface area are maximal) is the predominant factor. As such, extrapolations of PaCO_2_ values from ECO_2_ levels are problematic and large differences in lung aeration likely explain the large variability and difficulty in establishing a close relationship between PaCO_2_ and end-tidal CO_2_ levels in newborn infants [12], [13]. Indeed, low ECO_2_ levels are more likely to reflect poor lung aeration rather than over-ventilation during the immediate newborn period. Based on our data, determining whether a low ECO_2_ is due to poor lung aeration or over-ventilation can be determined by simply altering V_T_; an increasing ECO_2_ with a small increase in V_T_ indicates improving lung aeration and possible under-ventilation. However, following the immediate newborn period, after the lung has fully aerated, numerous other factors may contribute to altered ECO_2_ levels. In particular, low pulmonary blood flow, due to poor cardiac output or pulmonary hypertension resulting in left-to-right shunting through the ductus arterious, as well as alveolar gas trapping may reduce ECO_2_ levels.

In lamb studies, increasing V_T_ was closely associated with increasing ECO_2_ levels during lung aeration, following a sigmoidal shaped curve. As PEEP was constant, end-inflation lung volumes are mainly determined by V_T_, which explains the close relationship between V_T_ and ECO_2_ levels. After a V_T_ of ∼2 mL/kg, ECO_2_ levels increased linearly, indicating that at least 2 mL/kg of gas is required to initially penetrate into distal gas exchange regions. Although this largely reflects the dead space volume of the lung, it likely under-estimates this volume because many medium to small conducting airways, and the alveoli they supply, probably remained liquid-filled, as lung aeration is usually heterogeneous at birth [15], [16], [19]. After a V_T_ of ∼6 mL/kg, it appeared that increasing V_T_ only caused small increases in ECO_2_ levels, but care should be used in interpreting these data. As for rabbit kittens (Fig. 5), altering V_T_ in lambs changed ECO_2_ levels on an inflation-by-inflation basis (Fig. 2) even at higher V_T_ levels. However, a decrease in PaCO_2_ levels with time reduced the arterial/alveolar CO_2_ gradient causing a vertical shift in the relationship (Fig. 3B). This likely explains the flattening of the curve at higher V_T_ levels as larger volumes were mostly attained later in the experiment when PaCO_2_ levels were decreasing. Figure 3B, which shows data from a single animal, clearly demonstrates this concept. After 10 mins, when the V_T_ was held constant at 8 mL/kg, initially the maximum ECO_2_ was high but gradually decreased as the PaCO_2_ decreased (from 72.8 to 47.5 mmHg), causing a vertical shift in the data points.

It is interesting that CO_2_ clearance per inflation increased exponentially with increasing V_T;_ this measure is different to the maximum ECO_2_ value as it is also depends upon the duration of the expiratory gas flow. As a result, increasing V_T_ had little effect on CO_2_ clearance below a V_T_ of ∼6 mL/kg. Presumably, this is because expiratory gas flows are shorter and the dead space volume had a relatively greater impact on CO_2_ clearance at lower volumes, which became much less significant after ∼6 mL/kg. Although the precise factors regulating the relationship between CO_2_ clearance and V_T_ are unclear, as the inflation rate was kept constant it is interesting that the relationship was exponential and not linear. This likely results from the simultaneous contribution of other factors, in addition to increased surface area, to the efficiency of CO_2_ clearance. Simultaneous increases in pulmonary blood flow with lung aeration [3], [24], must greatly increase the efficiency of CO_2_ clearance, thereby contributing to the exponential relationship.

PC X-ray imaging of ventilated newborn rabbits allowed direct numerical comparisons between the degree of lung aeration and ECO_2_ levels. Consistent with data obtained from lambs, we found that end-inflation lung gas volumes directly related to ECO_2_ levels in the immediate newborn period, both during and after lung aeration. This is clearly evident in Movie S1 with the increase in ECO_2_ levels almost exactly coinciding with the first appearance of gas in the distal airways (see Fig. 4). The end-inflation gas volume of the lung at which ECO_2_ was first detected in preterm rabbit kittens was 3.4±0.3 mL/kg. Following subtraction of the calculated dead space volume, this indicates that ECO_2_ can be detected when only ∼7% (∼1.4 mL/kg) of the distal gas exchange regions of the lung are aerated.

Due to the small size (28.9±1.5 g) and V_T_ (∼0.2 mL) of preterm rabbits, it was not possible to incorporate a CO_2_ analyzer into the “Y” piece that connects to the ET tube to measure inflation-by-inflation changes in ECO_2_ levels. Instead, the CO_2_ analyzer (a main stream CO_2_ analyzer) was placed in the expiratory limb of the circuit, immediately downstream of the “Y” piece. As our ventilator does not use a bias gas flow [23], mainly expired gas enters this limb of the circuit. However, the combined dead space of the “Y” piece and CO_2_ analyzer (∼350 µL) was usually greater than the kitten’s V_T_. As a result, the ECO_2_ curve represents a smoothed diluted average of ECO_2_ levels across an entire inflation and the detection was slightly delayed. The delay was adjusted by time-shifting the ECO_2_ curve by the time required to expire 780 µL in preceding inflations (see Methods). Despite these limitations, we found that changes in ECO_2_ levels were extraordinarily sensitive to changes in end-inflation lung gas volumes (Fig. 6).

The parameter primarily regulating ECO_2_ levels in newborn rabbits was end-inflation lung volumes. Of the two components that comprise end-inflation lung volumes (FRC and V_T_; Fig. 6), only V_T_ was significantly related to ECO_2_ levels, as decreases in FRC were associated with both increases (Fig. 6, kitten 1) and decreases (Fig. 6, kittens 3 and 4) in ECO_2_ levels. When increases in ECO_2_ levels were associated with a decrease in FRC (Fig. 6, kitten 1), this was most likely due to a corresponding increase in V_T_ and an associated increase in CO_2_ clearance (Fig. 3). On the other hand, decreases in ECO_2_ levels were always associated with a decrease in FRC when end-inflation lung volumes (Fig. 6, kittens 1–4) also decreased. Movie S2 clearly demonstrates the effects of changing ventilation strategy on lung aeration, with the loss of PEEP and the decrease in V_T_ causing the basal lobes to either collapse or re-fill with lung liquid. In contrast, the apical lobes, particularly the right apical lobe (upper right of image), remained well ventilated and was possibly over-expanded (indicated by bulging between ribs), despite the remainder of the lung virtually collapsing at end-expiration.

Consistent with our findings in both lambs and rabbit kittens, we found that ECO_2_ levels were significantly associated with V_T_ in preterm infants, with some infants showing a strong relationship between these parameters (Fig. 8B). The large variability in the entire data set was likely due to large differences in ventilation success in these infants, as previously observed [8], as well as the effect that facemask or endotracheal leak will have on the measured ECO_2_ level [25]. For instance, in Figure 7 the initial mask ventilation failed to increase ECO_2_ levels and the infant’s heart rate remained low, despite good V_T_’s and no apparent mask leak. However, following intubation, smaller V_T_’s resulted in larger ECO_2_ levels, indicating that each inflation resulted in better lung aeration. This suggestion is consistent with the finding of a gradual increase in SpO_2_ and a rapid increase in HR (from 70 to 100 bpm between A and B) following intubation. In all preterm infants, we found that ECO_2_ levels increased above 10 mmHg approximately 28 (21–36) seconds before the HR increased above 100 beats/min. Thus, increasing ECO_2_ levels not only indicate the success and degree of lung aeration, they may also predict an impending increase in HR, although this is only preliminary data in a small number of infants (n = 10). It is not known why mask ventilation failed in the infant displayed in Figure 7, despite the measurement of good V_T_’s. However, it is possible that initially, when the airways are partially liquid filled, gas flows and volumes measured at the facemask do not necessarily reflect the flow of gas through the glottis and into the airways.

This study investigated whether ECO_2_ levels indicate the degree of lung aeration and provide useful feedback information when cardiorespiratory parameters fail to improve in response to assisted ventilation in the delivery room. We found that ECO_2_ levels are closely associated with end-inflation lung volumes during the immediate newborn period, preceding the increase in HR by ∼20 secs. Our data indicate that the surface area available for gas exchange is a major determinant of ECO_2_ levels during the newborn period. Although pulmonary perfusion and the concentration gradient for CO_2_ diffusion must also determine ECO_2_ levels at this time, the close relationship between end-inflation lung volumes and ECO_2_ levels indicates that ECO_2_ levels provide a good indication of aeration in gas exchange regions of the lung. As a result, ECO_2_ levels are unlikely to provide a reliable indication of PaCO_2_ levels in the immediate newborn period.

## Supporting Information

Movie S1 **Phase contrast X-ray movie of the increase in lung aeration in a newborn rabbit kitten (28 days of gestation).** The appearance of gas in the distal gas exchange units of the lung coincided with the increase in ECO_2_ (see Figure 4).
(MP4)

Movie S2 **Phase contrast X-ray movie of a newborn rabbit kitten (28 days of gestation) demonstrating the changes in lung aeration when ventilation parameters are altered.** The changes in ventilation parameters are demonstrated in Figure 6, Kitten 4.
(MP4)
