# Correction: Expired CO_2_ Levels Indicate Degree of Lung Aeration at Birth

**DOI:** 10.1371/annotation/44f67041-2f8e-42df-826a-82172ae05a22

**Published:** 2013-09-19

**Authors:** Stuart B. Hooper, Andreas Fouras, Melissa L. Siew, Megan J. Wallace, Marcus J. Kitchen, Arjan B. te Pas, Claus Klingenberg, Robert A. Lewis, Peter G. Davis, Colin J. Morley, Georg M. Schmölzer

Due to issues with the typesetting process, there were errors in the name of the sixth author.

The correct name of this author is: Arjan B. te Pas 

